# Supplementary material for: Empirical methods for controlling false positives and estimating confidence in ChIP-Seq peaks
Source: BMC Bioinformatics. 2008 Dec 5;9:523. doi: 10.1186/1471-2105-9-523 (PMC2628906; doi:10.1186/1471-2105-9-523)
Supplement: Additional File 1 — A variety of html documents from the USeq web site detailing the available applications, their best usage, output file type descriptions, command line menus, etc. [file 1471-2105-9-523-S1.zip › USeqUserGuides/JavaDoc.zip!/JavaDoc/bioroot/plasmid/PlasmidUploadBean.html]

PlasmidUploadBean


|  |  |  |
| --- | --- | --- |
| **Overview** \| **Package** \| **Class** \| **Use** \| **Tree** \| **Deprecated** \| **Index** \| **Help** | |  |
| **PREV CLASS**   **NEXT CLASS** | **FRAMES**    **NO FRAMES**     **All Classes** |  |
| SUMMARY: NESTED \| FIELD \| CONSTR \| METHOD | DETAIL: FIELD \| CONSTR \| METHOD |  |


---


## bioroot.plasmid Class PlasmidUploadBean

```
java.lang.Object
  bioroot.plasmid.PlasmidUploadBean
```

---

``` public class PlasmidUploadBean extends java.lang.Object ```

Plasmid bulk file upload bean.

**Author:**
:   nix

---

**Constructor Summary**

`PlasmidUploadBean()`


| **Method Summary** | |
| --- | --- |
| `java.lang.String` | `getFileName()` |
| `java.lang.String` | `getNotes()` |
| `PlasmidBean[]` | `getPlasmidBeans()` |
| `java.lang.String` | `getPlasmidReports()` |
| `boolean` | `isComplete()` |
| `void` | `setComplete(boolean b)` |
| `void` | `setFileName(java.lang.String string)` |
| `void` | `setNotes(java.lang.String string)` |
| `void` | `setPlasmidBeans(PlasmidBean[] beans)` |
| `void` | `setPlasmidReports(java.lang.String string)` |

**Methods inherited from class java.lang.Object**

`equals, getClass, hashCode, notify, notifyAll, toString, wait, wait, wait`

**Constructor Detail**

### PlasmidUploadBean

```
public PlasmidUploadBean()
```


**Method Detail**

### isComplete

```
public boolean isComplete()
```

---


### getFileName

```
public java.lang.String getFileName()
```

---


### getNotes

```
public java.lang.String getNotes()
```

---


### setComplete

```
public void setComplete(boolean b)
```

---


### setFileName

```
public void setFileName(java.lang.String string)
```

---


### setNotes

```
public void setNotes(java.lang.String string)
```

---


### getPlasmidBeans

```
public PlasmidBean[] getPlasmidBeans()
```

---


### getPlasmidReports

```
public java.lang.String getPlasmidReports()
```

---


### setPlasmidBeans

```
public void setPlasmidBeans(PlasmidBean[] beans)
```

---


### setPlasmidReports

```
public void setPlasmidReports(java.lang.String string)
```


---


|  |  |  |
| --- | --- | --- |
| **Overview** \| **Package** \| **Class** \| **Use** \| **Tree** \| **Deprecated** \| **Index** \| **Help** | |  |
| **PREV CLASS**   **NEXT CLASS** | **FRAMES**    **NO FRAMES**     **All Classes** |  |
| SUMMARY: NESTED \| FIELD \| CONSTR \| METHOD | DETAIL: FIELD \| CONSTR \| METHOD |  |


---
